# Characterization of Social Risk Factors Among Newborns Seen at an Urban Pediatric Primary Care Predictive of Appointment Nonattendance During the First 6 Months of Life

**DOI:** 10.1089/heq.2021.0053

**Published:** 2022-01-20

**Authors:** Brian Lefchak, Ann Cushwa, Hans Kersten, Kelly Courts, Katie McPeak

**Affiliations:** ^1^ Drexel University College of Medicine, Philadelphia, Pennsylvania, USA.; ^2^ Center for the Urban Child and General Pediatrics, St. Christopher's Hospital for Children, Philadelphia, Pennsylvania, USA.

**Keywords:** pediatric, newborn, social risk, appointment, screen, primary care

## Abstract

**Purpose:** Appointment attendance is critical in monitoring health and well-being of children. Low income Medicaid-insured families with newborns often experience social risks that may affect attendance. This project sought to characterize social risk factors present at first newborn visits predictive of future appointment nonattendance.

**Methods:** Retrospective cohort study of minority and Medicaid-insured population at St. Christopher's Hospital for Children using a standardized social risk screener administered at first newborn visits as part of routine clinical care. In total, 720 survey responses between December 2016 and June 2017 were correlated with electronic health record-derived sociodemographic and appointment attendance data in the first 6 months of life. Nonattendance included missed and canceled appointments. Caregiver-reported social risk factors were included as covariates in linear regressions predicting proportion nonattendance outcomes.

**Results:** Newborn caregivers identified many social risk factors including mental health diagnoses (14%), lack of child care support (45%), and food insecurity (9%). Approximately 74% had nonattendance with 41% missing or canceling a quarter or more appointments. Number of siblings (*p*<0.01) and maternal age (*p*<0.01) were most predictive for nonattendance, respectively. Other social risks were not significant except for maternal mental health (*p*=0.01) among those identifying number of risk factors above cohort average (16%).

**Conclusion:** Screening of newborns at first medical visits can be used to characterize social risks. Most social risk factors at first visits were not strongly predictive of nonattendance, although our results suggested associations between non-attendance and maternal demographics, mental health and household makeup.

## Introduction

Approximately 28 million children in the United States live in families with low incomes, increasing the likelihood of experiencing social risks and poor health outcomes.^1–3^ Well-child check (WCC) appointments are crucial interventions for monitoring childhood outcomes and providing medical care, screening, and guidance.^4–7^ Pediatric providers are often challenged to identify and address all social risks due to time and resource constraints as well as appointment nonattendance.^3,8^

Nonattendance of medical appointments is a serious and widespread reality associated with negative impacts on patient health outcomes, medical costs, office productivity, and the quality of patient–provider interactions.^9–11^ Previous studies have posited associated factors including prior no shows, distance to travel, lower socioeconomic status (SES), insurance, race/ethnicity, age, use of medical interpreters, and others.^9,12^

Newborn WCCs are critical for establishing a medical home and providing anticipatory guidance.^7,13^ Low-income Medicaid-insured newborns have been at known risk for delays in access to WCCs that can impact subsequent care.^13^ Although literature exists regarding social risk factor screening and appointment nonattendance in the pediatric population under 1 year of age, relatively less is well characterized among newborns and their families at first visit appointments.^8,14–16^ Many studies have retrospectively screened patients after missed appointments, but fewer studies have made use of screening before the nonattendance, and even fewer if any regarding newborns specifically.^9,17^

Given the effects of social risk factors on appointment nonattendance and childhood health outcomes, our study objective was to characterize social risks present at first newborn visits associated with future appointment nonattendance in the first 6 months of life. This study took place in Philadelphia that has one of the highest rates of urban childhood poverty in the United States and where many families live in impoverished neighborhoods that propagate various socioeconomic disadvantages.^18,19^

Our hypothesis is that social risk factors identified during the first visit are associated with increased appointment nonattendance. Therefore, an earlier recognition of these risk factors may assist clinical practices in preventing delays in care during a vulnerable phase of childhood development.

## Methods

### Setting

**FIG. 1.**
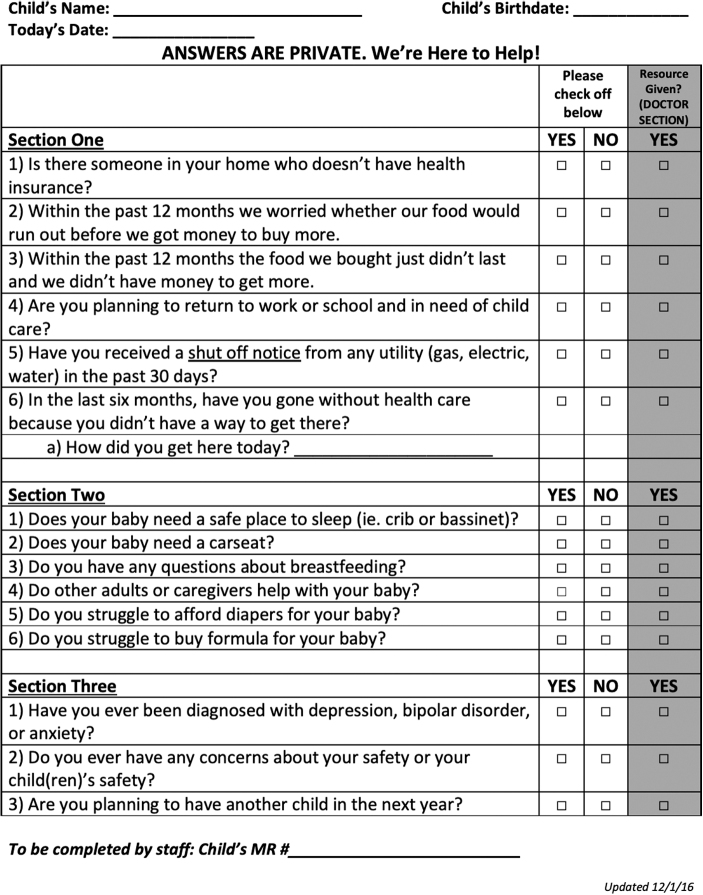
Newborn social risk screener.

St. Christopher's Hospital for Children (SCHC) is located in one of the poorest sections of Philadelphia.^19^ The Center for the Urban Child (CUC) at SCHC serves as an academic primary care medical home and houses a newborn clinic that provides focused medical care and includes relevant wraparound services, such as an on-site lactation consultant and designated social worker. Beginning in December 2016, mothers of newborns initiating medical care at the CUC were asked to complete a standardized social risk screening survey at their first visit, which typically occurs within the first week of life (Fig. 1).

The survey is modeled on a Bright Futures and WE CARE (Well-child Care Visit, Evaluation, Community Resources, Advocacy, Referral, Education) instrument and collects information about social risks and needs that may necessitate a social work consult for referrals and additional support.^3,15^ Forms are available in both English and Spanish to fit the needs of the patient population. The survey is distributed by medical assistants at rooming as a self-administered paper questionnaire and reviewed by the provider during the visit.

### Sample

Approximately 150 newborns are seen monthly (1700/year) at SCHC and on average 87% of their caregivers were screened during the study timeframe. The study sample included all patients whose caregivers completed social needs surveys from December 1, 2016 through June 30, 2017, a total of 794 patients. All data were manually entered into Microsoft^®^ Excel spreadsheets on secure HIPAA-compliant devices by social work staff. Sociodemographic and appointment attendance data were abstracted from the electronic health record (EHR) NextGen^®^.

In total, 74 survey responses were unable to be matched to records and excluded from study. Appointment data were abstracted for all visits up to 6 months of age, defined by our study as 168 days using the Pennsylvania Early and Periodic Screening, Diagnosis and Treatment Program as a guideline. Six months of life was chosen due to availability of data retrieval at the time of study.

### Design

Abstracted data included visit date and status as WCC or non-WCC and missed/no show, and rescheduled or canceled. A no show was defined as missing a scheduled visit with no notice. A canceled appointment was defined as missing a scheduled visit with prior notice but without rescheduling. Appointment nonattendance was defined as either a no show or a canceled visit. All appointment attendance outcomes included both WCC and non-WCC visits. The number of scheduled visits was defined by the total aggregate of encounters available from EHR data to capture patients' actual encounters with health care. The primary outcome reported was proportion nonattendance.

Social risk factors were measured using 13 categorical binary (yes/no) caregiver-reported survey item responses (Fig. 1) with an affirmative response being defined as a positive social risk factor regarding health insurance (survey item 1.1), food insecurity (1.2 and 1.3), child care (1.4), utility payment (1.5), transportation (1.6), safe newborn sleep space (2.1), car seat access (2.2), single caregiver support (2.4), newborn supplies (2.5 and 2.6), maternal mental health (3.1), and child safety (3.2). Descriptive statistics of social needs were calculated using the 13 categorical survey item responses, 3 categorical sociodemographic EHR items (newborn gender, insurance, and race/ethnicity), and 2 numerical sociodemographic EHR items (number of siblings and maternal age).

These variables were ultimately included in our study as they represent social risk externalities related to nonattendance identified elsewhere in the literature.^7,9^ Survey items 1.6a (user response to transportation modality), 2.3 (questions regarding breastfeeding), and 3.3 (planning to have another child) were excluded due to limited response rate and study applicability. Incomplete survey responses were limited to <0.8% missing at least one item and excluded from analysis. Descriptive statistics and association analyses were conducted using Statistical Program for Social Sciences^®^ (SPSS) through univariate, bivariate, and linear regression models, with a two-sided *p*-value <0.05 indicating statistical significance.

Linear regression models were constructed for the primary study outcome proportion nonattendance. The primary regression included all study variables of interest from survey and sociodemographic data in the entire cohort (*n*=720). Two additional linear regressions were constructed for secondary exploratory analyses. The second regression was constructed excluding a large proportion of patients with complete attendance (*n*=190) to identify differences in appointment nonattendance between the entire cohort and those with any nonattendance.

A third regression was constructed for the subgroup of respondents with three or more survey risk factors (*n*=117). This was of particular interest as these respondents reported more social risks than the cohort average. In all linear regressions, automated backward elimination process entailed sequential removal of variables by SPSS to the least number of variables without statistical loss of model fit. Standardized beta coefficients (*β_s_*) were calculated to facilitate comparison of effect size across variables.

There was minimal risk from study participants and all aspects were approved by the Drexel University Institutional Review Board.

## Results

### Cohort demographics

Of the 720 patients in the sample (Table 1), the combined majority had caregiver-identified race/ethnicity as African American or Hispanic (43% and 36%, respectively), and almost all subscribed to Medicaid insurance plans (95%). Race/ethnicity, insurance, and newborn gender demonstrated no significant association with other variables. The mean maternal age of the cohort was 26 years old, with 89% aged 20 years or older. Newborns on average had two siblings, with 24% having three or more.

**Table 1.** Demographic Characteristics of Study Sample

Total number of completed surveys	720
Maternal age	
Mean	26
Range	14–43
Age 19 years or younger	77 (11%)
Age 20 years or older	643 (89%)
Insurance plans	
Medicaid	687 (95%)
Private	33 (5%)
Race/ethnicity	
African American, non-Hispanic	313 (43%)
Hispanic	258 (36%)
Caucasian, non-Hispanic	44 (6%)
Asian, non-Hispanic	19 (3%)
Multiracial	23 (3%)
Other or unknown	63 (9%)
No. of siblings	
Mean	2
Range	0–10
Newborns with three or more siblings	175 (24%)
Newborns with two or less siblings	545 (76%)
Appointment attendance	
Mean total scheduled	8
Range	2–18
Mean proportion nonattendance	20%
Range	0–82%

### Patterns of social risks

**FIG. 2.**
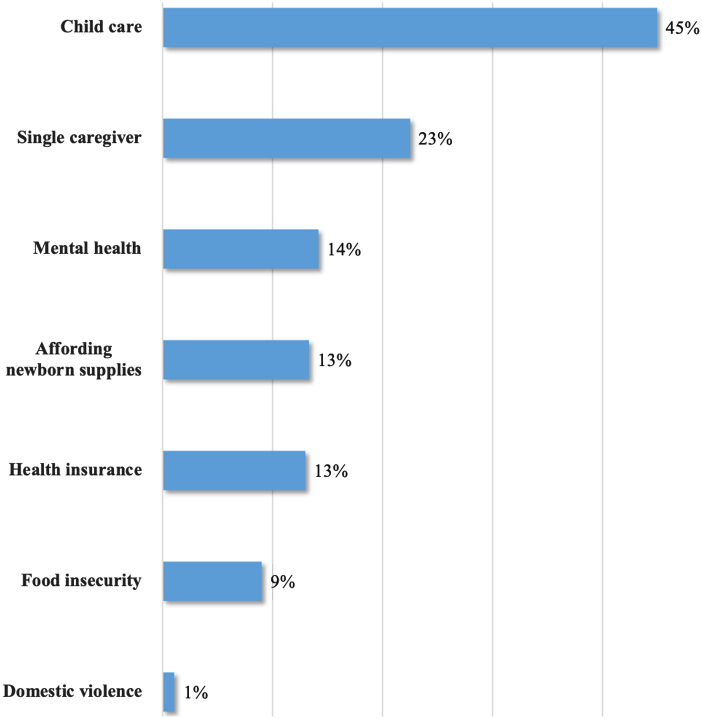
Social risks identified by newborn caregivers (*n*=720).

The most common psychosocial need from the survey was child care among those planning to return to work or school (45%). More than 70% of caregivers reported at least one risk (range 0–7); 28% reported none, and 16% identified three or more. Only 1–4% of caregivers indicated a need for safe newborn sleep space or car seat, difficulty affording utilities, transportation concerns, or addressing concerns regarding child safety (Fig. 2).

Among caregivers identifying any risks on the intake survey (*n*=521), the mean number identified was 2. Food insecurity demonstrated the largest association with additional risks, as caregivers who identified food insecurity also identified three additional risk factors on average (*p*<0.01). Most risks were not strongly correlated with other survey items (Spearman correlation values ranging from 0.33 to −0.08).

### Appointment nonattendance

**FIG. 3.**
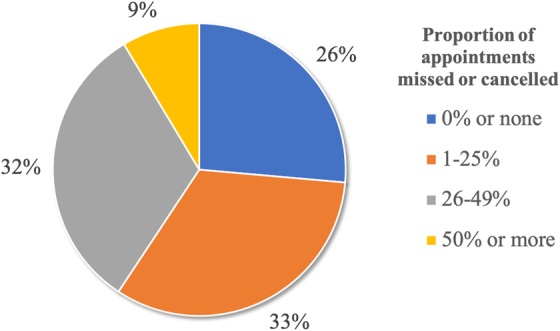
Proportion appointment nonattendance among the cohort (*n*=720).

On average, infants had eight scheduled appointments in the first 6 months of life and the mean proportion of appointment nonattendance was 20% (Fig. 3). Twenty-six percent of the cohort attended all appointments, whereas 41% missed or canceled a quarter or more of their scheduled appointments. The only variables from survey and sociodemographic data shown to be significantly associated with nonattendance rates greater than the cohort mean were maternal mental health and maternal age 19 years or less. Maternal mental health diagnosis and maternal age 19 years or less were nearly twice as prevalent among caregivers with nonattendance above the cohort mean compared with those without any nonattendance (chi-square statistics 18% versus 9%, *p*=0.02, and 14% versus 8%, *p*=0.02).

### Linear regression analyses

Three linear regression models included all study variables of interest and consisted of one primary and two secondary exploratory analyses (Table 2). The primary regression of the entire cohort (*n*=720) identified number of siblings (β*_s_*=0.22, *p*<0.01) and maternal age (β*_s_*=−0.13, *p*<0.01), respectively, as the main covariates for nonattendance.

**Table 2.** Linear Regression Models for Appointment Nonattendance

	Total cohort (***n***=720)		Any appointment nonattendance (***n***=530)		Three or more social risk factors (***n***=117)	
	***β_s_***	***p***	***β_s_***	***p***	***β_s_***	***p***
No. of siblings	0.22	<0.01	0.24	<0.01	—	—
Young maternal age	–0.13	<0.01	–0.11	0.03	—	—
Single caregiver	—	—	0.08	0.09	—	—
Mental health diagnosis	—	—	—	—	0.25	0.01

The overall distribution of appointment data was skewed due to a sizable proportion (26%) of patients with complete attendance. Therefore, a second regression was restricted to only those with appointment nonattendance (*n*=530). Like the primary analysis, number of siblings (β*_s_*=0.24, *p*<0.01) and maternal age (β*_s_*=−0.11, *p*<0.01) were identified as the main predictive factors for additional nonattendance. In this analysis, single caregiver status (β*_s_*=0.08, *p*=0.09) also trended toward significance.

Although the entire patient population represented in our study is likely to experience social disadvantages, ∼16% of our sample reported three or more social risk factors on the intake survey that was above the cohort average. To evaluate contributors to nonattendance among those with increased self-reported risk burden, a third regression was constructed for this subgroup of respondents (*n*=117). In this small and highly selected subsample, none of the previously identified predictors was significant. Rather, maternal mental health diagnosis (β*_s_*=0.25, *p*=0.01) was identified as the predictive factor for nonattendance.

## Discussion

This study evaluated social risk factors impacting appointment attendance during the first 6 months of life at an urban academic practice. Despite many reported social risk factors found to be nonpredictive, our early screening of newborns at the first visit indicates associations between non-attendance and maternal demographics, mental health and household makeup.

### Social risk factors

Our survey instrument was modeled after a 2007 study by Garg et al. that demonstrated the feasibility of a single caregiver-reported screening for multiple social risk factors at an urban pediatric clinic.^3,15^ Although our study employed a similar screening method of a comparable predominantly minority, lower SES, and Medicaid-insured population, we examined social risk factors among newborns at the first outpatient visit that had been excluded from the 2007 study surveying families with older children (average age 3.4 years old).^3^

The most common risk factor from our survey was lack of child care among those planning to return to work or school. The 2007 Garg et al. study on which we modeled our survey reported a 28.6% prevalence of this need among caregivers in the screening intervention group.^3^ Our results, however, suggested a greater prevalence (45%) among caregivers with younger pediatric patients. This difference may be due to the novel and time-intensive demand of newborn care among newer and lower SES caregivers and is consistent with literature citing parental support as an important priority during perinatal appointments.^20^

Our study uncovered many risk factors among families with newborns. Caregivers identified consistent needs for child care, food assistance, health insurance, and newborn supplies. Many of these social risks have been shown to be prevalent among lower SES families.^1–3^

### Appointment nonattendance

Infants in our study demonstrated significantly higher nonattendance than other comparable studies.^9,12,21^ A 2015 study by Samuels et al. demonstrated a 20.4% no-show rate among older pediatric patients (average age 7.6 years old) and inferred older age as one factor associated with nonattendance.^9^ A 2011 study by Van Berckelaer et al. of lower SES infants <2 years of age found that WCC adherence may in fact drop further after the sixth month of life particularly.^21^ The study hypothesized that caregivers may simply prioritize care of younger infants perceived as more vulnerable.^21^

Notably, although our study population shared many similar demographics regarding race/ethnicity, insurance, and SES with both of these studies, we uncovered patterns suggesting greater infant nonattendance.^9,21^ Unlike Van Berckelaer et al., our study design included total encounters of both WCCs and non-WCCs, which we consider a unique strength that is more representative of patients' actual health care utilization while controlling for similar social risks. Our study showed that ∼74% of our total cohort had some degree of nonattendance with 41% missing or canceling a quarter or more of their appointments. Such implications for patient care may not be apparent based on WCC timeframe attendance alone.

### Predictors of nonattendance

Although our study found some associations, many variables were not strongly predictive of appointment nonattendance. These included factors that have been associated with pediatric nonattendance elsewhere, such as insurance, transportation difficulties, and various SES metrics.^9,12,14^

There are several possibilities for this lack of association. Foremost, “colocated nonmedical services” can impact appointment attendance.^4^ Our finding may in fact reflect the degree to which the newborn clinic wraparound services are able to address unmet social needs, which, in turn, may encourage subsequent appointment attendance. In the Van Berckelaer et al. study, lower income was found to be predictive of appointment adherence perhaps due to such eligibility for insurance and other services. Our study, however, did not have access to income and other data abstraction to comment on this specifically.^21^

Other contributors may include the size or relative homogeneity of our sample. This in part prompted a secondary analysis of individuals with the most self-reported risk burden that demonstrated that maternal mental health combined with other social risks was predictive of future visit nonattendance. Interestingly, caregivers with mental health diagnoses did not report additional risk factors on average, suggesting a salient independent effect. We also found mental health diagnoses to be more prevalent among caregivers with the most nonattendance, although studies on this association have been mixed, ranging from adequate WCC attendance to increased hospital utilization.^22,23^

Maternal mental health has been known to impact many pediatric outcomes and could potentially exhaust caregivers' abilities to attend appointments while contending with multiple other stressors.^24–26^ Providers should utilize dedicated screening for perinatal depression and related mood disorders to prevent nonattendance, particularly if multiple psychosocial stressors are present.^23,26^

Our results collectively suggest that younger single caregivers with other children were most likely to miss or cancel future appointments. Despite the decline of teen pregnancy rates historically, many are still multigravida.^26,27^ Young often teenage caregivers stand to benefit from approaches that validate their experiences as outlined by the American College of Obstetricians and Gynecology recommendations.^26,28^ Caregiver support, despite being a prevalent cohort need in our study, only trended toward significance among those with nonattendance, suggesting having another caregiver at home for younger caregivers may buffer specifically against future nonattendance to some small effect.

These findings may support programs such as postnatal home visits.^29^ Although regression effect sizes were small, these particular findings are similar to those from Van Berckelaer et al. that showed WCC adherence was predicted by maternal marital status and number of children.^21^ Our results likewise suggest that demographic factors such as maternal age and household makeup should be strongly considered by clinical practices seeking to prevent nonattendance.

### Limitations

There are several limitations to our study. Although our survey was developed by experienced clinical staff and in part modeled after a validated screening instrument, our survey itself was not a validated tool.^3,15^ Furthermore, although this survey screened a substantial number of families for many relevant social risk factors for the purposes of clinical and social staff work, not all patients seen were screened nor all relevant social risk factors screened related to newborns or nonattendance.

Our survey data demonstrated high rates of completion and medical staff were available for assistance, nevertheless there is the possibility caregivers misunderstood or did not feel comfortable completing survey questions. Furthermore, our survey did not differentiate among type or severity of maternal mental health diagnoses.

Our study design was limited to infants' first 6 months of life and a combined measure of total appointments. Although it is indeed possible that increased number of sick visits, follow-up, or immunization appointments are related to or may result from missed or canceled WCCs and other visits, our results did not further differentiate among appointment types or evaluation of care received elsewhere due to limited availability of data abstraction. Finally, the generalizability of our study may be limited as our population consisted of almost universally Medicaid-insured and minority race/ethnicity patients from a single site.

## Conclusion

Infants of lower SES caregivers demonstrate high rates of social risk factors and overall appointment nonattendance. Prospective social risk screening of newborns at first medical visits may be predictive for subsequent nonattendance. Although many social risk factors were identified in this newborn population, most were not predictive of nonattendance or strongly associated with those expected at most risk. Our early screening of newborns, however, suggests associations between non-attendance and maternal demographics, mental health and household makeup.

Younger single caregivers with other children or caregivers with mental health diagnoses and multiple stressors are at higher risk for nonattendance. Understanding trends among newborn–caregiver dyads may assist clinical practices in tailoring upstream resources for patients at highest risk for nonattendance to promote equitable health outcomes. Future study could expand on effects of social risks including mental health, nonattendance variance across appointment types, and interventions to address reported caregiver needs at additional life cycle points.

## Abbreviations Used

CUC: Center for the Urban Child
EHR: electronic health record
SCHC: St. Christopher's Hospital for Children
SES: socioeconomic status
SPSS: Statistical Program for Social Sciences
WCC: well-child check
